# A Meta-Path-Based Prediction Method for Human miRNA-Target Association

**DOI:** 10.1155/2016/7460740

**Published:** 2016-09-15

**Authors:** Jiawei Luo, Cong Huang, Pingjian Ding

**Affiliations:** ^1^College of Information Science and Electronic Engineering & Collaboration and Innovation Center for Digital Chinese Medicine of 2011 Project of Colleges and Universities in Hunan Province, Hunan University, Changsha, Hunan 410082, China; ^2^College of Information Science and Electronic Engineering, Hunan University, Changsha, Hunan 410082, China

## Abstract

MicroRNAs (miRNAs) are short noncoding RNAs that play important roles in regulating gene expressing, and the perturbed miRNAs are often associated with development and tumorigenesis as they have effects on their target mRNA. Predicting potential miRNA-target associations from multiple types of genomic data is a considerable problem in the bioinformatics research. However, most of the existing methods did not fully use the experimentally validated miRNA-mRNA interactions. Here, we developed RMLM and RMLMSe to predict the relationship between miRNAs and their targets. RMLM and RMLMSe are global approaches as they can reconstruct the missing associations for all the miRNA-target simultaneously and RMLMSe demonstrates that the integration of sequence information can improve the performance of RMLM. In RMLM, we use RM measure to evaluate different relatedness between miRNA and its target based on different meta-paths; logistic regression and MLE method are employed to estimate the weight of different meta-paths. In RMLMSe, sequence information is utilized to improve the performance of RMLM. Here, we carry on fivefold cross validation and pathway enrichment analysis to prove the performance of our methods. The fivefold experiments show that our methods have higher AUC scores compared with other methods and the integration of sequence information can improve the performance of miRNA-target association prediction.

## 1. Introduction

 MicroRNAs (miRNAs) are important endogenous 21-22 nt RNAs that play important regulatory roles in gene expression. Several studies have shown that miRNAs participate in the regulation of amount cellular process, such as cell proliferation and differentiation [1], development [2], and disease [3, 4]. Considering the importance of miRNAs, it is critical to identify and decipher miRNA-target interactions at a genome level.

All the time, scientists and academics have made great efforts in uncovering the associations between miRNA and its targets by using biological experiments [5–8]. However, it is impossible to depict a complete picture of miRNA regulation mechanisms only relying on biological experiments due to the high expenses on time and cost [9]. Therefore, computational approaches must be designed to be a cost-effective choice to describe the complete mechanism of miRNA regulatory. Now, many computational approaches show great advantage in predicting putative miRNA targets [10–13].

Over the past decade, plenty of miRNA-mRNA pairs prediction approaches have been developed to identify miRNA targets by using sequence data, including TargetScanS/TargetScan [14, 15], miRanda [16], Pictar [17], DITAT-MicroT [18], and PITA [19]. The majority of these prediction algorithms were built on specific binding rules, including the degree of site conservation, thermodynamic stability, sequence complementarity, energy, target site context, secondary structure, and site accessibility. Because of the complex character of miRNA-target interactions, these sequence-based methods have relatively high false-positive rate [20]. Furthermore, those predictions methods were mostly only at static sequence level, leading to those exact interactions that are specific to certain conditions or diseases. More importantly, sequence-based methods do not support statistically significant predictions as the miRNA binding sites are small, causing the results by different methods to be inconsistent.

To identify condition-specific interactions, many methods integrating expression profiles information into sequence-based predictions have been proposed to study miRNA-mRNA regulatory mechanism. These methods are based on the assumption that gene has negative correlations with the miRNA because of the downregulation effect that miRNAs have on their targets. These methods can be divided into four categories including simple correlation analysis [21, 22], simple/regularized regression models [23–25], Bayesian inference [19, 26], and causally inference between miRNAs and their targets [27]. Pearson correlation, one of the typical simple correlation methods, is commonly used in computing the strength of the association between a pair of miRNA and mRNA. However, Pearson correlation has high false-positive rate as the simplicity of it. Furthermore, Pearson correlation is mainly used in predicting linear associations. Lasso regression [24, 25], one of the regression models, is a high-dimensional method used to extract more reliable association as they usually optimize the network provided by sequence-based method and retain the relatively reliable edges. GenMir++ [19], the first and well-cited Bayesian inference method, calculates the existence probabilities of the relationship between a miRNA and its target based on a Bayesian model. However, this method needs prior information, such as sequence information. In general, methods in Bayesian category assume different priors [28] and are difficult in learning parameters. MCMG (joint analysis of multiple cancer for MiRNA-gene interactions), based on empirical Bayesian model [29], identifies miRNA-target associations that are either specific to a cancer type or common to several cancers by jointly analyzed across cancers. Muniategui et al. use do-calculus to estimate the causal effects the miRNA have on all the target mRNAs. The four categories methods can improve prediction performance as they integrate expression profiles information into sequence-based prediction methods [30]. But, most of the existing approaches cannot effectively use the valuable experimentally validated information [31–34]. Besides, the lack of miRNA expression profile may cause the unreliability of the predicted miRNA-target associations.

On the whole, the limitations of existing methods are summarized as follows. Firstly, sequenced-based prediction algorithms suffer from a high false-positive rate; second, the methods integrating expression profile data can only analyse one cancer every time; third, some methods cannot effectively utilize validated knowledge. To solve these problems, we propose two network-based approaches, RMLM and RMLMSe, to identify miRNA-target interactions based on meta-path. Meta-path is a good measuring method to compute the relatedness between the same or different types of objects in heterogeneous information network, as it contains a certain sequence of different link types [35]. Different meta-paths have different semantic meaning corresponding to different relationships between connected objects. In RMLM, we first utilize RM (a meta-path related measure proposed by Cao et al. [36]) to evaluate the existence probability of a link between miRNA and its targets. As different meta-path corresponds to different relation graphs, we may improve the final performance when integrating these different graphs by appropriate weights corresponding to different meta-paths. Thus, we then employ logistic regression and maximum-likelihood estimation (MLE) method to estimate the weight of different meta-path. Here, the issue of relationship prediction can be regarded as a two-class classification problem by using Bayesian analysis and logistic regression and then the MLE method can be employed to estimate the parameter vector. In RMLMSe, sequence information is integrated to improve the performance of the RMLM. Furthermore, as global approaches, RMLM and RMLMSe can remodel the missing relationship for all the diseases-associated miRNAs at the same time. Fivefold cross validations, pathway enrichment analysis about global network, and three important diseases network show that our proposed methods work well in predicting the relationship between miRNA and its target.

## 2. Problem Definition

In this part, we describe the concepts of Heterogeneous Information Network and meta-path used in this paper.

### 2.1. Heterogeneous Information Network

A heterogeneous information network is an important type of information network with multiple types of nodes and multiple types of links [36–38]. It can be represented as *G* = (*V*, *E*). *V* is the set of nodes, which involves *n* types of nodes: *V*_1_ = {*v*_1_^1^, *v*_1_^2^,…, *v*_1_^*x*^},…, *V*_*n*_ = {*v*_*n*_^1^, *v*_*n*_^2^,…, *v*_*n*_^*y*^}, where *v*_*i*_^*j*^ is *j*th node of type *i*. *E*⊆*V* × *V* is the set of links between the nodes in *V*, which involves *m* types of links.

**Figure 1:**
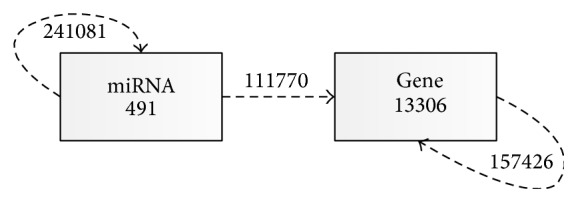
Network schema of the miRNA-target network. The network contains two types of objects, miRNA and its targets. Each box represents one type of nodes, and each dashed line represents one type of links. The numbers in the figure represent the numbers of nodes/links of different types.

Each type of links between source node of type *i* and target node of type *j* corresponds to a binary relation *R*_*ij*_. More specifically, *R*_*ij*_^*st*^ = 1 if *v*_*i*_^*s*^ (*s*th nodes of type *i*) and *v*_*j*_^*t*^ (*t*th nodes of type *j*) are connected by a link of type *R* ^*ij*^. For example, in Figure 1, the relation between miRNA and gene is “regulate.” Particularly, *R*_*ij*_^*st*^ equals 1 if *s*th miRNA regulates *t*th gene.

Moreover, a weighted matrix *W*_*ij*_ = |*V*_*i*_| × |*V*_*j*_| can be used to describe the relation *R*_*ij*_, where *W*_*ij*_^*st*^ ∈ [0,1] is the existence probability of link between nodes *v*_*i*_^*s*^ and *v*_*j*_^*t*^. Particularly, *W*_*ij*_^*st*^ = 1, if there exists an edge between *v*_*i*_^*s*^ and *v*_*j*_^*t*^. Otherwise, *W*_*ij*_^*st*^ is set as 0 in initialization for the unknown links.

### 2.2. Meta-Path

In heterogeneous information network, meta-path is defined on network schema. A meta-path *P* is described in the form *A*_1_ → *A*_2_ → ⋯*A*_*n*−1_ → *A*_*n*_, where *A*_*i*_ is *i*th type of object and a relation must exist from *A*_*i*−1_ to *A*_*i*_,  *i* = 2,3,…, *n*. Similarly, we define the inverse path of *P* as *P* ^−1^, denoted as *A*_*n*_ → *A*_*n*−1_ → ⋯*A*_2_ → *A*_1_. Specifically, relation A_*i*−1_ → *A*_*i*_ is the inverse relation of *A*_*i*_ → *A*_*i*−1_. For example, in Figure 1, a meta-path “gene → miRNA → gene” is a composite sequence between genes. The relation from miRNA to gene is “regulate” and the relation from gene to miRNA is “regulate^−1^”; “regulate^−1^” is the inverse relation of “regulate.” Meta-path can connect object of the same or different types; thus, they can show knowledge between homologous objects or heterologous objects. For example, in Figure 1, for gene *i* and gene *j*, they can connect through another gene *k*, gene  *i* → gene  *k* → gene  *j*; this means gene *i* and gene *j* have relation with gene *k* simultaneously and there may exist relation between gene *i* and gene *j* by information transfer. However, gene *i* and gene *j* can also connect by miRNA *k*, gene  *i* → miRNA  *k* → gene  *j*; this means gene *i* and gene *j* are regulated by a common miRNA *k* and there may exist relation between gene *i* and gene *j* by information transfer. Different meta-paths of different relations correspond to different relation graphs with different semantics. For example, in Figure 1, the meta-path “gene → gene” denotes that two genes are connected by “PPI” links, while the meta-path “gene → miRNA → gene” corresponds to the semantic that two genes are regulated by a common miRNA. Thus, similarity between the same or different type of nodes can be described by different meta-paths with different semantics.

In this paper, the meta-path from source node of type *i* to target node of type *j* is described as *P*_*ij*_. Particularly, *P*_*ii*_ is the meta-path between nodes of the same type *i*; *P*_*iis*_ is *s*th meta-path of *P*_*ii*_. *P*_*jj*_ and *P*_*jjt*_ are the same to *P*_*ii*_ and *P*_*iis*_. *P*_*ijst*_ is a meta-path by connecting *P*_*iis*_, *R*_*ij*_, and *P*_*jjt*_ in sequence; it can be written as a certain sequence of relations: *R*_*k*_0_*k*_1__, *R*_*k*_1_*k*_2__,…, *R*_*k*_*n*−1_*k*_*n*__; here *k*_0_ = *i*, *k*_*n*_ = *j* and the length of *P*_*ijst*_ is *n*.

## 3. Method

RMLM and RMLMSe consist of three steps. In the first step, we utilize MISIM (proposed by Wang et al. in [39]) to calculate the miRNA functional similarity matrix and then construct the heterogeneous network. Next, we calculate the relatedness between any miRNA and its targets and extract the feature vector of these interactions. In RMLM, the feature vector only contains different relatedness of different meta-path between miRNA and its targets. However, in RMLMSe, the feature vector not only contains different relatedness from different meta-path, but also contains feature extracted from sequence information. Finally, logistic regression and MLE method are employed to compute the different weights of different meta-paths. Sections 3.1–3.4 are the detailed introduction of RMLM.  Section 3.5 is about RMLMSe.

### 3.1. Construction of the Heterogeneous Network

#### 3.1.1. miRNA-miRNA Similarity Estimation

In [39], Wang et al. compute miRNA-miRNA functional similarity score based on the assumption that miRNAs with similar functions tend to be related to similar disease. To get the miRNA-miRNA similarity matrix, there contains three procedures. We take miRNA *i* and miRNA *j* as an example. First, we identify diseases that related to these two miRNAs, encoded as *D*_*i*_ and *D*_*j*_. We can obtain the relationship between miRNAs and diseases from The Human MicroRNA Disease Database (HMDD dataset). Then, we can calculate similarity of any pair of diseases using a hierarchical structure. The semantic similarity of disease is calculated based on directed acyclic graph obtained from the US National Library of Medicine in 2015 (MeSH, https://www.nlm.nih.gov/mesh/). Finally, we utilize the similarity score between *D*_*i*_ and *D*_*j*_ to compute the relatedness score between miRNA *i* and miRNA *j*. In this paper, we use SM (a 491 × 491 matrix) to represent the miRNA-miRNA similarity matrix; SM(*i*, *j*) is the functional similarity score between miRNA *i* and miRNA *j*.

#### 3.1.2. Construction of the Heterogeneous Network

We construct the heterogeneous network by connecting the miRNA interaction network and PPI utilizing the bipartite graph of the miRNA-target association network. The schema of the heterogeneous network used in this paper is illustrated in Figure 1. The network contains two types of objects, miRNA and its targets. A meta-path *P* is defined at the object type level and is denoted in the form of *A*_1_ → *A*_2_ → ⋯*A*_*n*−1_ → *A*_*n*_, where *A*_*i*_ represent the object of type.

### 3.2. Relatedness Measure

The RM measure [36] is a path-constrained measure and it can calculate the relatedness of heterogeneous objects with the same or different types in a uniform framework. It has been proven that RM has some good properties, such as symmetric and self-maximum, and has shown its potential to mining valuable information in heterogeneous network. Therefore, here we use RM measure to calculate the relatedness between miRNA and its targets. RM measure is based on the Linkage Homophily Principle defined as follows.

*Linkage Homophily Principle*. Two nodes are more likely to be directly linked if most of their respective similar nodes are linked.

In general, the computing of nodes similarity is based on their neighbors. However, in heterogeneous networks, the same type similar nodes can be linked by heterogeneous nodes through composite paths. For example, two similar genes can be connected by a common miRNA, “gene → miRNA → gene.” Thus, we can utilize meta-path to extract the generalized neighbor and define the similarity. Here, we first extract the meta-path that connects the source node and target node. We take source node *v*_*i*_^*p*^ and meta-path *P*_*iis*_ as an example. The neighbors of node *v*_*i*_^*p*^ based on *P*_*iis*_ are the nodes of type *i* that linked to *v*_*i*_^*p*^ by *P*_*iis*_, denoted as *N*_*i*_^*p*^. Similarly, we can get the generalized neighbors of target node *v*_*j*_^*q*^ and meta-path *P*_*jjt*_, denoted as *N*_*j*_^*q*^. Then, we can use the connectivity between *N*_*i*_^*p*^ and *N*_*j*_^*q*^ to calculate the link's existence probability between nodes *v*_*i*_^*p*^ and *v*_*j*_^*q*^.

Suppose RMP_*iis*_ is the similarity matrix of *i*th type node along the meta-path *P*_*iis*_. Similarity, RMP_*jjt*_ represents the similarity matrix of *j*th type node along the meta-path *P*_*jjt*_. In general, similarity can be calculated by the path counts. Expected path number is the number where all of the links may exist from node of type *i* to node of type *j*. Let meta-path *P*_*ijst*_ = {*R*_*k*_0_*k*_1__, *R*_*k*_1_*k*_2__,…, *R*_*k*_*n*−1_*k*_*n*__}, *k*_0_ = *i*, and *k*_*n*_ = *j*; then the expected path number RMP_*ijst*_ is computed as follows:

(1) $$\begin{array}{c} RMP_{ijst}=\overset{n}{\underset{p=1}{\prod }}w_{k_{p-1}k_{p}}=RMP_{iis}\times W_{ij}\times RMP_{jjt}. \end{array}$$

Here, *P*_*ijst*_ is a meta-path composed of *P*_*iis*_, *R*_*ij*_, and *P*_*jjt*_; RMP_*ijst*_ is a matrix whose size is |*V*_*i*_| × |*V*_*j*_|. The computation of RMP_*iis*_ (or RMP_*jjt*_) is similar to the computation of RMP_*ijst*_.

Now the relatedness between nodes of type *i* and nodes of type *j* along the meta-path *P*_*ijst*_ can be formulated as follows:

(2) RMijstRMPijstRMPiis×1×RMPjjt=RMPiis×Wij×RMPjjtRMPiis×1×RMPjjt.

Here** 1** is a matrix in which all the elements are 1 and the size of is |*V*_*i*_| × |*V*_*j*_|. Similarly, RM_*ijst*_ is also a |*V*_*i*_| × |*V*_*j*_| matrix and RM_*ijst*_^*pq*^ is the relatedness measured between *v*_*i*_^*p*^ and *v*_*j*_^*q*^ following *P*_*ijst*_.

### 3.3. Construction of the Feature Vector

We can get the relatedness between miRNAs and their targets as described in Section 3.2. Now we get the feature vector as follows:

(1) Extract meta-path *P*_*ii*_ of *i*th type node and *P*_*jj*_ of *j*th type node.
(2) Compute the similarity based on any pair of meta-paths *P* ^*ii*^ and *P* ^*jj*^ and then get the feature vector.

In RMLM, the feature vector between miRNA *i* and gene *j* is defined as

(3) $$\begin{array}{c} \phi_{ij}=\left(\;f_{1},f_{2},\ldots ,f_{n}\;\right), \end{array}$$

where *f*_1_ to *f*_*n*_ represent the different similarities of different meta-paths with different semantic meaning.

### 3.4. Parameter Estimation

As different meta-path corresponds to different relation graphs, the final result may be improved by combining these different graphs through different weights. Here, logistic regression and maximum-likelihood estimation (MLE) method can be employed to estimate the weight.

In this paper, we regard the issue of relationship prediction as a two-class classification problem by using Bayesian analysis and logistic regression. Based on logistic regression and under general assumption [31, 32], the posterior probability of a specific relation can be formulated as follows:

(4) pxi=1 ∣ φi,ω=exp⁡ωTφiexp⁡ωTφi+1,

(5) pxi=0 ∣ φi,ω=1exp⁡ωTφi+1.

Here *ω* is a weight vector served as parameters and *φ*_*i*_ is the feature vector of the link *x*_*i*_. Then, MLE method can be employed to estimate the parameter vector *ω*. The likelihood function can be written as

(6) $$\begin{array}{c} L\left(\;\omega ;x_{1},x_{2},\ldots ,x_{N}\;\right)=\overset{N}{\underset{i=1}{\prod }}p\left(\;x_{i}\;|\;\varphi_{i},\omega \;\right). \end{array}$$

Here *x*_*i*_ is the link to calculate and *N* is the number of links, *φ*_*i*_ is the feature vector that is calculated according to RM, and *ω* is the weight vector of the feature according to different meta-path. The log likelihood of (6) is

(7) ln⁡Lω;x1,x2,…,xN=∑i=1NxiωTφi−ln⁡1+exp⁡ωTφi.

The log likelihood (7) is a convex function [40]. Hence, we can find a unique global optimal solution by solving a convex optimization problem.

### 3.5. Final Score

The logistic regression based algorithm returns a set of posterior probabilities. One can directly use those probabilities to make decision. However, the posterior probabilities do not always work well because it is difficult to set a threshold for a relation between miRNA and its target. Here, we utilize a percentage value as the final score to evaluate the strength of the relation between a miRNA and its target. The final score is calculated as follows:

(8) $$\begin{array}{c} q_{i}=\frac{\left|\left\{j\;|\;p_{i}\geq p_{j}\right\}\right|}{n},\;i=1,2,\ldots ,n. \end{array}$$

Here {*p*_1_, *p*_2_,…, *p*_*n*_} is the posterior probabilities of any association, and *q*_*i*_ is the top percentage value of *p*_*i*_ among all those posterior probabilities. The larger the final score is, the more likely the association exists.

### 3.6. Integration of Sequence Information

In RMLMSe, we integrate sequence information to improve the performance of the RMLM. Here, we use sequence information from database TargetScan, miRanda, and PITA. As they have a relatively high false-positive rate, we only download conserved targets information and select the data whose Pct > 0.9 from TargetScan, mirSVR > 0.6 from miRanda, and data in PITATOP to improve the reliability of the regulation relationships. Sequence information from these databases acts as new features in feature vector used in RMLMSe. Taking interaction between miRNA *i* and gene *j* as an example, its feature vector can be written as

(9) $$\begin{array}{c} \phi_{ij}=\left(\;f_{1},f_{2},\ldots ,f_{n},f_{m},f_{m+1},f_{m+2}\;\right). \end{array}$$

Here *f*_1_ to *f*_*n*_ represent the different feature of different meta-paths and *f*_*m*_, *f*_*m*+1_, and *f*_*m*+2_ represent the feature of sequence information from TargetScan, miRanda, and PITA, respectively.

### 3.7. Algorithm

The process description of RMLM and RMLMSe is given as follows.

*Input*. The disease set *d*_*i*_ of each miRNA *i* from HMDD and DAG *g*_*j*_ of each disease *j* from MeSH, the protein interaction matrix SP, and the miRNA-protein matrix MP.

*Output*. The vector of final score for each unknown interaction between miRNA and its targets.

(1) Calculate the miRNA-miRNA functional similarity matrix SM as described in Section 3.1.1.
(2) Extract meta-path *P*_*ii*_ of *i*th type node and *P*_*jj*_ of *j*th type node. We set the max length of meta-path between the same type node as (3).
(3) Concatenate *P*_*iis*_ (*s*th meta-path of *P*_*ii*_), *R*_*ij*_, and *P*_*jjt*_ (*t*th meta-path of *P*_*jj*_) in sequence to compose a meta-path *P*_*ijst*_ going from the source nodes of type *i* to target nodes of type *j*. Then, the relatedness between miRNA and its target based on meta-path *P*_*ijst*_ is calculated according to (2).
(4) Calculate the different similarity of different meta-path and get the feature vector of each interaction. The feature vectors used in RMLM and RMLMSe are described in Sections 3.3 and 3.5.
(5) Estimate parameters *ω* by maximizing the log likelihood ln⁡*L*(*ω*; *x*_1_, *x*_2_,…, *x*_*N*_) in (7) based on *x*_*i*_ and *φ*_*i*_, *x*_*i*_ is the link to be calculated, and *N* is the number of links.
(6) Calculate the probability for each unknown interaction according to (4) by using *ω* and feature vector.
(7) Calculate the final score according to (8).

## 4. Results

### 4.1. Datasets

*The Human MicroRNA Disease Database.* HMDD [41] provides a comprehensive resource of experimentally verified miRNA-disease associations. We can get the information through a website at http://www.cuilab.cn/hmdd. The database (in June 2014) contains 5100 associations between 491 miRNAs and 326 diseases. In this paper, we first analyse the global network. Then, we analyse another three diseases, Ovarian Neoplasms (OV), Lung Neoplasms (Lung), and Breast Neoplasms (Breast). The miRNAs associated with OV, Lung, and Breast are 114, 132, and 202, respectively.

*The Protein-Protein Interaction Database.* The PPI network was constructed by combining DNA-protein data from TRANSFAC [42] and protein interaction data obtained from Bossi and Lehner [43], respectively. The database contains 13306 proteins and 157426 interactions between proteins.

*Experimentally Validated miRNA-mRNA Interaction Databases*. The posttranscriptional regulatory knowledge is obtained from miRNA-target database miRTarbase* v6.1*. When mapping onto our miRNA-target matrix, it retains 111770 interactions. We can get the information through a website at (http://mirtarbase.mbc.nctu.edu.tw/).

*Predicted miRNA-mRNA Interaction Database.* We also utilize sequence information in database TargetScan* v7.0*, miRanda released at 2010, and PITA* v6*. These databases are available online at http://www.targetscan.org/, http://www.microrna.org/, and http://genie.weizmann.ac.il/pubs/mir07/, respectively.

### 4.2. Comparisons with Other Methods

**Figure 2:**
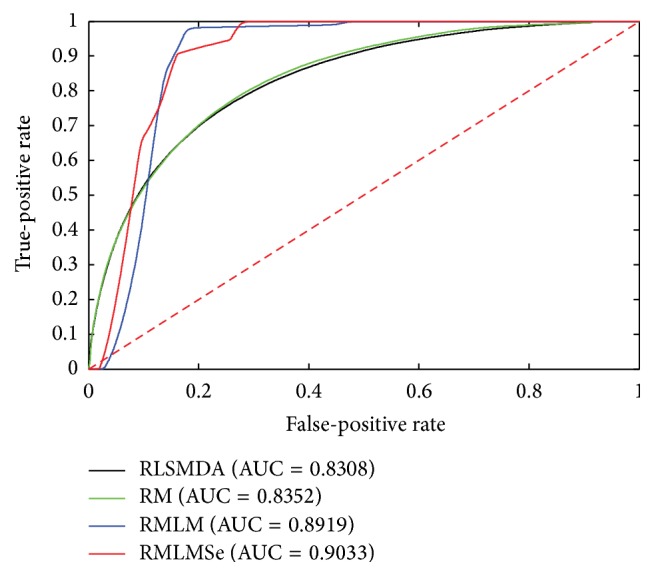
The ROC curve of the global network.

**Figure 3:**
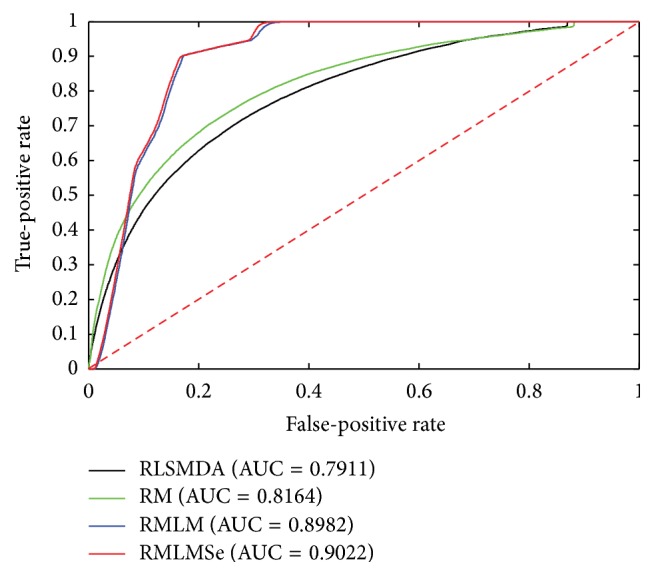
The ROC curve of the OV network.

**Figure 4:**
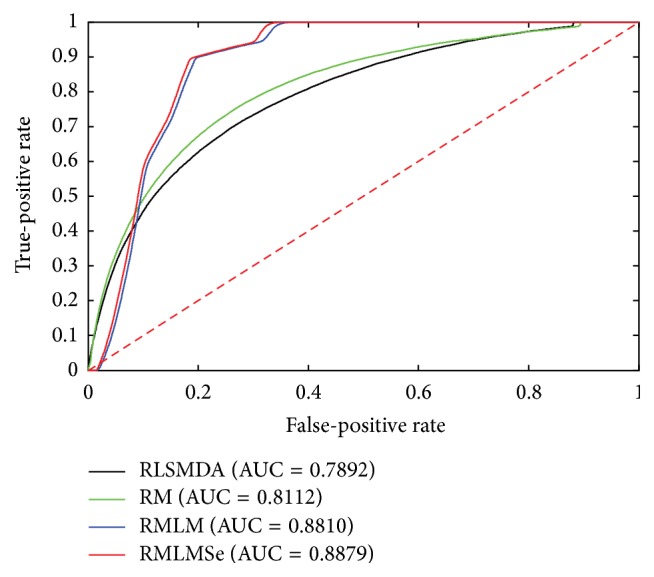
The ROC curve of the Lung network.

**Figure 5:**
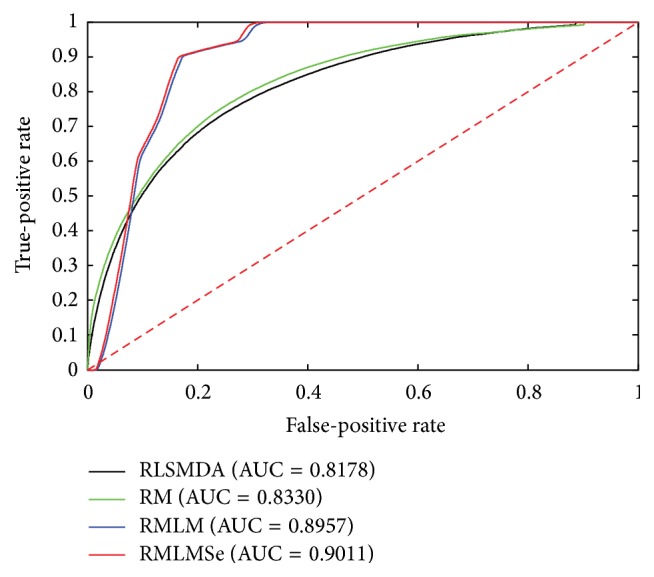
The ROC curve of the Breast network.

To compare the performance of RMLM and RMLMSe, we applied RLSMDA [44] and RM [36] to the same testing data. RLSMDA was introduced to predict disease-miRNA association. We encoded RLSMDA in MATLAB according to the derivation process of the authors. Here, we set *ω* used in RLSMDA as 0.5. RM was implemented in MATLAB with source code available from authors personal homepage. RM is the measurement used to calculate the similarity of objects in heterogeneous networks. Here, the sum of the different similarities corresponding to different meta-paths is utilized to predict the miRNA-gene associations. All experiments are carried on a Windows 7 professional computer (Inter(R) Xeon(R) CPU, 2.93 GHz, 56 G RAM, 64-bit OS). The performance of each method is evaluated by fivefold cross validation. First, all known miRNA-target associations were split into five sets of the same size randomly: one set was set aside as the test set and the other four sets were used as train sets. The experiment was repeated five times so that each set was hidden once and each hidden miRNA-target pair obtained a predict relevance score. The ROC (receiver operating characteristic) curve was calculated according to the various TPR (true-positive rate) and the various FPR (false-positive rate) through a varying threshold. The area under the ROC curve (AUC) is employed to show the overall performance of methods. We can see from Figure 2 that RMLM and RMLMSe always work better than RLSMDA and RM. There is only slight improvement when sequence information is employed, where the AUC score increases from 0.8919 to 0.9033. This may have two reasons. First, the performance of the RMLM already achieves a very high AUC score and there is only a little room for it to be further improved by using additional prior information. Second, the amount of the sequence information mapped onto the miRNA-target matrix is little; for example, when TargatScan, miRanda, and PITA mapped onto the miRNA-target matrix, they leave 16,7403, 10,4631, and 13,7229 interactions, about 1.6~2.6% of the entire size of the miRNA-target matrix MP (a 491 × 13306 matrix). Although the improvement of the sequence information is not significant, the increased AUC score still indicates that additional knowledge is helpful for improving the prediction performance as any prior knowledge, such as sequence information, Go Ontology annotations, gene copy numbers, and gene methylation, related to miRNA-target associations can be employed to predict associations. Figures 3, 4, and 5 are the result when we execute the methods on OV, Lung, and Breast database, respectively. The results are similar to Figure 2. RMLM and RMLMSe always work better than RLSMDA and RM, and RMLMSe only have a slight improvement than RMLM.

### 4.3. The Number of Links Predicted by Our Methods

Here, we present the number of interactions predicted based on different thresholds in RMLM and RMLMSe. As shown in Table 1, the numbers of interactions predicted in RMLM are higher than in RMLMSe among all of the threshold. This can further indicate the performance improvement in RMLMSe. In future, we can utilize the associations predicted by our method to construct miRNA-target regulatory network and extract regulatory modules and hub nodes.

### 4.4. Functional Validation of mRNAs

When we get the result of the global dataset, we compute every mRNA score and extract the top 250 mRNAs to carry on the pathway enrichment analysis with the focus on KEGG (Kyoto Encyclopedia of Genes and Genomes) pathways (adjusted *p* value < 0.05). In this paper, *p* value calculated by hypergeometric test is a statistical value that represents the significant enrichment of pathways. The smaller the *p* value is, the more significant the pathway enrichment is. As shown in Table 2, many of the KEGG pathways are highly related to many cancers and respective biological process, for instance, glioma, prostate cancer, and colorectal cancer. Furthermore, pathways in cancer are closely related to many cancers and P53 signaling pathways is proved to be related to the processes of cell division and DNA replication [45]. The result of Lung KEGG pathways is shown in Table 3. The pathway focal adhesion [46], adherens junction [47], and ErbB signaling pathway [48] are proved to be related to Lung.

## 5. Discussion and Conclusion

The rapid increase of various biological data provides challenges and opportunities for us to complete the global miRNA regulatory mechanism. In recent years, academics have made great efforts to predict miRNA targets. However, each method has its pros and cons, and the performance of a method varies on different datasets. Thus, how to get precise results is a long-time challenge for miRNA-target association prediction.

In this paper, two novel methods, RMLM and RMLMSe, were developed. In RMLM, we first construct miRNA-miRNA similarity matrix. Second, we use RM to evaluate the different relatedness between miRNAs and its target based on different meta-path and extract the feature vectors of links; different meta-path corresponds to different relation graphs; we can improve the performance by combining these different graphs through different weights of corresponding meta-paths. Third, logistic regression and MLE method were employed to estimate the weight. Here, the issue of relationship prediction is regarded as a two-class classification problem by using Bayesian analysis and logistic regression and then MLE method can be employed to estimate the parameter vector. Then, we estimate the posterior probabilities between miRNAs and its targets based on the feature vectors of links and the corresponding parameter vectors. Finally, the final scores are obtained by using the percentage values of individual posterior probabilities. In RMLMSe, we utilize more information such as sequence information from TargetSacn, miRanda, and PITA to improve the performance of the RMLM. The results showed that there are slight improvement when sequence information is integrated.

Compared with other methods, RMLM and RMLMSe proposed by us have higher AUC scores. Besides, we conduct pathway enrichment analysis and found many relevant pathways. These results indicate that our two methods were reasonable and credible.

The comparison results of RMLM and RMLMSe indicate that our methods have the capability to integrate more biological data, such as sequence data and gene copy number. Thus, with the rapid growth of the gene regulatory knowledge, our method can integrate more prior information to improve the prediction performance.

In addition, disease target inference [49, 50], disease-miRNA prioritization [51–54], and lncRNA-disease association prediction [55] are also the immediate areas of research focus to further study therapeutic strategy. Due to the scalability of the proposed methods, RMLM and RMLMSe could be applied to the different constructed heterogeneous networks to infer disease target, miRNA-disease association, and lncRNA-disease association, respectively. Moreover, the performance of our methods should be further evaluated after extending.

Of course, RMLM and RMLMSe also have some limitations that need to be improved in the future. Firstly, our methods utilize the network topology and known miRNA-gene associations to calculate the relatedness between miRNA and its target. It may cause bias to miRNA-gene pair which has more neighbor nodes. Furthermore, although the better performance is obtained by our methods on the whole, the predictive results should be further improved, especially for the small output. In the future, the prediction performance will be further improved by integrating more reliable biological data and obtaining more known miRNA-gene associations.

## Figures and Tables

**Table 1:** The number of links predicted by our methods based on different thresholds.

Database	Methods	Validated	Th ≥ 0.9	Th ≥ 0.8	Th ≥ 0.7	Th ≥ 0.6	Th ≥ 0.5
Global	RMLM	11,1770	17,2912	20,4894	23,4327	26,5883	79,8049
	RMLMSe	11,1770	17,6625	21,0909	24,2946	28,1782	80,7688

OV	RMLM	4,2730	5,3683	5,9580	6,4676	6,9759	23,3784
	RMLMSe	4,2730	5,3891	5,9954	6,5526	7,1565	23,4562

Lung	RMLM	4,7764	5,8511	6,4339	6,9397	7,4816	24,5323
	RMLMSe	4,7764	5,8870	6,4881	7,0437	7,9293	24,6261

Breast	RMLM	6,4403	8,6555	9,8883	10,9659	12,0730	36,4375
	RMLMSe	6,4403	8,6690	9,9540	11,1719	12,6556	36,6573

The “validated” column is the number of links validated in database miRTarbase *v6.1* and “Th” represents the threshold.

**Table 2:** In RMLMSe, the enrichment KEGG pathways of global dataset.

	Enrichment KEGG pathways	*p* value
1	p53 signaling pathway	4.27*E* − 10
2	Chronic myeloid leukemia	8.80*E* − 10
3	Bladder cancer	3.24*E* − 09
4	Glioma	6.03*E* − 09
5	Melanoma	1.35*E* − 08
6	Pathways in cancer	2.34*E* − 08
7	Prostate cancer	1.01*E* − 07
8	Cell cycle	1.61*E* − 07
9	Small cell lung cancer	9.71*E* − 07
10	Pancreatic cancer	3.26*E* − 06

The *p* values have been obtained through hypergeometric test.

**Table 3:** In RMLMSe, the enrichment KEGG pathways of lung dataset.

	Enrichment KEGG pathways	*p* value
1	p53 signaling pathway	5.15*E* − 10
2	Pathways in cancer	3.11*E* − 08
3	Small cell lung cancer	1.12*E* − 06
4	Non-small cell lung cancer	1.04*E* − 05
5	Focal adhesion	1.53*E* − 05
6	Neurotrophin signaling pathway	1.81*E* − 04
7	Adherens junction	6.05*E* − 04
8	ErbB signaling pathway	1.34*E* − 03
9	Pathogenic *Escherichia coli* infection	1.89*E* − 03
10	MAPK signaling pathway	1.31*E* − 02

The *p* values have been obtained through hypergeometric test.
